# The Application of Omics Technologies to Study Axon Regeneration and CNS Repair

**DOI:** 10.12688/f1000research.17084.1

**Published:** 2019-03-20

**Authors:** Andrea Tedeschi, Phillip G Popovich

**Affiliations:** 1 Department of Neuroscience and Discovery Themes Initiative, College of Medicine, Ohio State University, Columbus, Ohio, 43210, USA; 2 Center for Brain and Spinal Cord Repair, Institute for Behavioral Medicine Research, Ohio State University, Columbus, Ohio, 43210, USA

**Keywords:** axon regeneration, brain injury, spinal cord injury, epigenomics, transcriptomics, kinomics, phosphoproteomics, metagenomics

## Abstract

Traumatic brain and spinal cord injuries cause permanent disability. Although progress has been made in understanding the cellular and molecular mechanisms underlying the pathophysiological changes that affect both structure and function after injury to the brain or spinal cord, there are currently no cures for either condition. This may change with the development and application of multi-layer omics, new sophisticated bioinformatics tools, and cutting-edge imaging techniques. Already, these technical advances, when combined, are revealing an unprecedented number of novel cellular and molecular targets that could be manipulated alone or in combination to repair the injured central nervous system with precision. In this review, we highlight recent advances in applying these new technologies to the study of axon regeneration and rebuilding of injured neural circuitry. We then discuss the challenges ahead to translate results produced by these technologies into clinical application to help improve the lives of individuals who have a brain or spinal cord injury.

## Introduction

Central nervous system (CNS) trauma causes permanent disability, imposing huge economic and emotional burdens on affected family and society. No therapies exist that will effectively restore function to individuals who have an injury to the brain or spinal cord. Recovery of brain and spinal cord functions in adults might be achieved by promoting axon sprouting and regeneration, either alone or in combination with other promising approaches such as neuroprotection ^1– 3^, cell reprogramming and transplantation ^4– 8^, brain–computer interface and epidural stimulation ^9– 14^. In the injured adult mammalian CNS, however, axon sprouting and regeneration are limited and this is due in part to both the poor intrinsic regenerative potential of adult CNS neurons ^15, 16^ and the hostile cellular and molecular environment that develops at the site of injury ^17– 21^. These are major obstacles that must be overcome to effectively promote axon regeneration, sprouting, and functional recovery after CNS trauma ^22– 24^. Recent data indicate that it is possible to overcome such barriers. For example, it is feasible to reprogram adult mammalian neurons into a growth-competent state and remove extracellular growth inhibitors to promote regrowth of axons that project to the brain and spinal cord ^16, 25– 32^.

Here, we review the most recent data, emphasizing how omics technologies are improving our insight into novel mechanisms that regulate axon regeneration and also the feasibility of rebuilding functional neuronal circuits after CNS injury. We also discuss the challenges to applying these new discoveries in the clinic to maximize recovery of function.

## Omics approaches to study spinal cord injury

**Figure 1.**
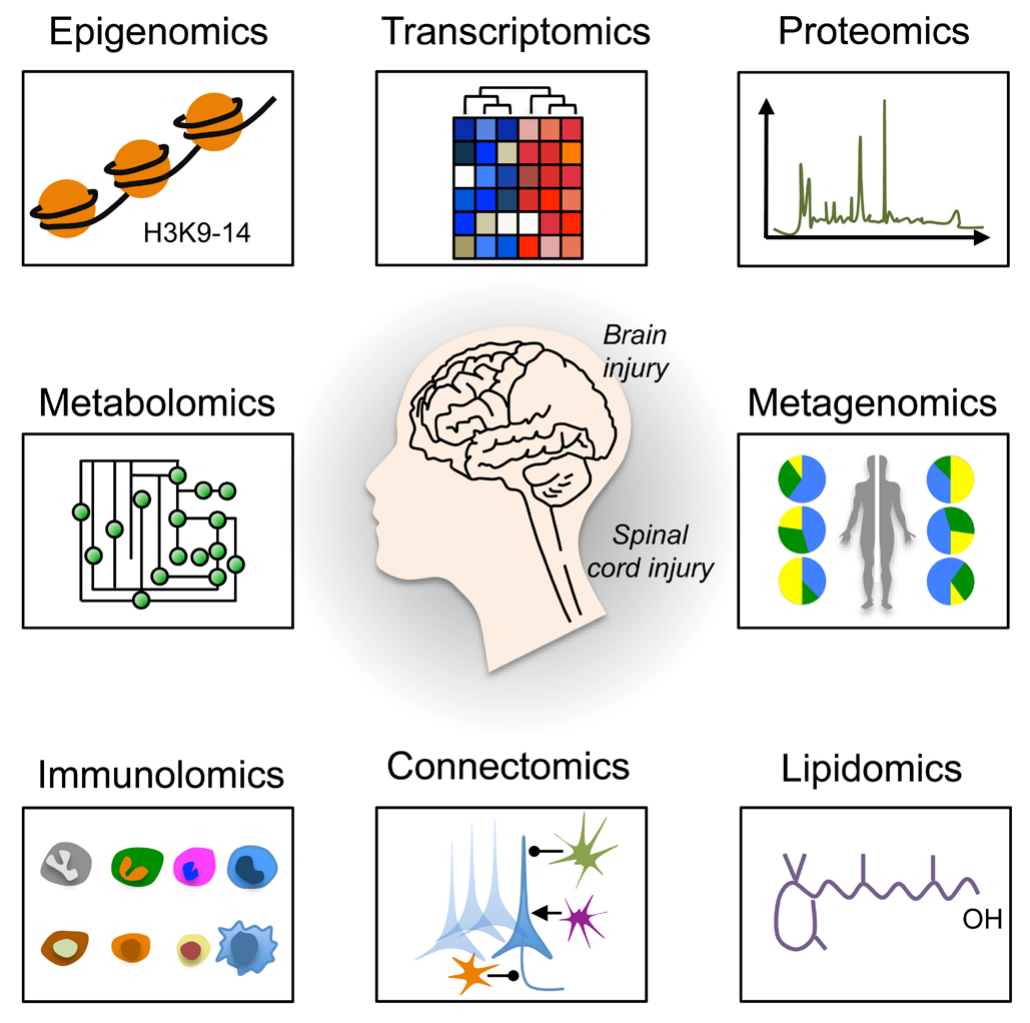
A multi-layer omics approach to study axon regeneration. A schematic representation of omics approaches to study axon regeneration is shown. Epigenomics studies epigenetic modifications on DNA or histone proteins that alter gene expression. DNA methylation and histone modifications are the most well-characterized epigenetic modifications. Transcriptomics examines the transcriptome that comprises all RNA transcripts (for example, mRNA and non-coding RNAs) in a given cell population. New technologies, including RNA sequencing at single-cell resolution, have been developed that allow the identification of genes and transcript variants that are actively expressed, co-expressed, or repressed. After epigenomics and transcriptomics, proteomics represents the next step in the study of any given biological system. Indeed, proteomics is the large-scale study of the proteome (for example, the set of proteins produced in an organism, system, or biological context). Protein activity is also regulated by many different factors in health and disease other than the gene expression level. Modern high-throughput technologies allow the investigation of protein location, turnover, post-translational modification, activity, and interactions in depth. Phosphoproteomics represents a branch of proteomics that focuses entirely on the identification and characterization of phosphorylated proteins. Metabolomics is the study of substrates and products (also called metabolites) of cellular metabolism and their interactions within a biological system. Each cellular activity is reflected by the presence of specific metabolites. Therefore, metabolomics represents a powerful approach to study the state and phenotype of any given biological system. Metagenomics is the genomic analysis of microbial communities from environmental and biological samples, such as the gut microbiota. Indeed, metagenomics allows the study of intestinal microbiome diversity and dysbiosis as well as its relationship between human health and disease. Immunolomics profiles cells of the immune system, antibodies, and cytokine responses in a comprehensive manner. With the advent of powerful imaging methods and molecular and genetic tools, it is now possible to create comprehensive maps of connections within the nervous system. Connectomics refers to the production and study of such connections and the molecular interactions that pair cells. One of the emerging fields of biomedical research is certainly lipidomics. Lipidomics is the large-scale study of cellular lipids at both the structural and functional levels.

High-throughput omics technologies, including epigenomics, transcriptomics, proteomics, metabolomics, metagenomics, immunolomics, connectomics, and lipidomics have revolutionized the way we study brain and spinal cord injury (SCI) ^27, 33– 41^ ( Figure 1). When combined with high-content screening ^42– 44^, data from these technologies are providing unprecedented insight into how an injury to the brain or spinal cord affects the roles and interrelationships of various genes, molecules, cells, and body systems. As a result, novel targets and pathways are emerging as critical regulators of effective axon growth as well as regeneration and remodeling of both injured and spared neural circuits. In this review, we will focus on a subset of new omics data, using examples from epigenomics, transcriptomics, kinomics, phosphoproteomics, and metagenomics studies. We will then discuss how these technologies have helped identify novel biological mechanisms, such as neuronal metabolism and mitochondria transport, that contribute to axon regeneration failure.

### Epigenomics

Recent data indicate that the induction of regenerative gene expression, a prerequisite for activating axon growth programs, relies partly on creating a more permissive chromatin environment in the nucleus of injured neurons ^45– 51^. Epigenomic screens of adult dorsal root ganglia (DRG) neurons injured by a peripheral nerve lesion (PNL), an experimental condition that switches DRG neurons into a regenerative-competent state, identified Tet methylcytosine dioxygenase 3 (Tet3) as a critical regulator of axon growth and regeneration. After PNL, Tet3 is upregulated along with the epigenetic mark 5-hydroxymethylcytosine (5hmC) in DRG neurons ^52^. By oxidizing 5hmC, Tet3 reverses DNA methylation. Interestingly, epigenomic mapping in DRG neurons after injury to the peripheral (regenerative) or central (no regenerative effect) projecting axons triggered differential 5hmC changes that were associated with distinct signaling pathways ^52^. Nearly half of the genes that were differentially regulated after peripheral lesion contain 5hmC alterations ^52^, suggesting that 5hmC is a previously unrecognized mechanism that controls the regenerative potential of injured neurons.

Although transcriptional events that turn on the expression of regeneration-associated genes are recognized as important steps in the activation of cell-autonomous regeneration programs ^53^, far less is known about how gene inactivation affects these programs. A recent study identified ubiquitin-like containing PHD ring finger 1 (UHRF1)-dependent DNA methylation as a critical epigenetic mechanism responsible for silencing expression of genes that are required to promote axon regeneration in DRG neurons ^54^. After PNL, a decrease in miR-9 causes a transient increase in the expression of the RE1-silencing transcription factor (REST) and UHRF1 ^54^. During embryogenesis, REST acts as master regulator by inhibiting the expression of many neuronal genes ^55^. While a transient increase in REST primes injured DRG neurons for enhanced axon regeneration, UHRF1 interacts with DNA methyltransferases and methyl groups on histone H3, creating epigenetic marks that silence promoter elements of tumor suppressor genes such as the phosphatase and tensin homolog ( *PTEN*) and *REST* ^54^. Since sustained expression of REST in neurons is known to cause axon guidance defects ^56^, UHRF1-dependent epigenetic silencing may be required to fine-tune REST activity and thus axon regeneration programs. Together, these data support the idea that neurons may need to revert to an immature or intermediate state to successfully unlock developmental programs for axon regeneration ^57– 62^.

### Transcriptomics

Advanced transcriptomics analyses have identified several genes and gene networks that regulate axon regeneration success and failure ^27, 34, 63, 64^. However, the efficiency and success of translation of these genes have received less attention. By ribosome pull-down and metabolic isotopic labeling, a recent study analyzed gene translation and protein synthesis within the regeneration-associated program in DRG neurons. Of the proteins that undergo *de novo* synthesis in regenerating DRG neurons, apolipoprotein E (ApoE), which has been previously implicated in axon growth and regeneration ^65– 68^, is one of the most robustly synthesized proteins ^69^. DRG neurons cultured in the presence of an ApoE receptor inhibitor extend shorter neurites, providing evidence that neuronal ApoE is an autocrine regulator of axon growth ^69^. It is likely, though speculative, that ApoE facilitates recycling of cholesterol from degenerating axons for integration into new membranes during the process of axon regeneration. Alternatively, cholesterol may be synthesized in the cell body and then efficiently delivered to the axonal compartment via anterograde transport of lipid-containing vesicles. **

An unbiased genome-wide loss-of-function screen in cerebral cortical projection neurons *in vitro* identified Rab27b, a member of the Rab subfamily of GTPases, as a cell-autonomous factor that restricts axon regeneration ^39^. Adult worms lacking Rab27 exhibit greater regeneration of GABA neurons. Moreover, optic nerve regeneration, raphespinal sprouting, and locomotor recovery all are enhanced in mice lacking Rab27 ^39^. Interestingly, *Caenorhabditis elegans* Rab27 mutants have defects in synaptic transmission ^70^. Given that Rab27 localizes in synaptic-rich regions and participates in the transport of synaptic vesicles ^71^, removing or blocking Rab27 in adult neurons may promote axon regeneration by shifting the trafficking of new cell membrane from synapses to the axolemma. Indeed, new membrane insertion is necessary for axon elongation ^72^.

Interestingly, data from an independent study show that selective exclusion of Rab11 vesicles, which are needed for axon elongation, contributes to axon regeneration failure. Rab GTPases coordinate vesicle trafficking ^73^, thereby allowing growth-promoting cargoes to be delivered to the axon. In cultured rat cortical neurons, overexpressing Rab11 decreases axon retraction and augments new growth cone formation and enhanced axon regeneration occurs in an integrin-dependent manner ^74^. It is likely that changes in spatiotemporal interaction between Rab GTPases and specific guanine nucleotide exchange factors contribute to diversify the role of Rab GTPases in axon growth and regeneration.

**Figure 2.**
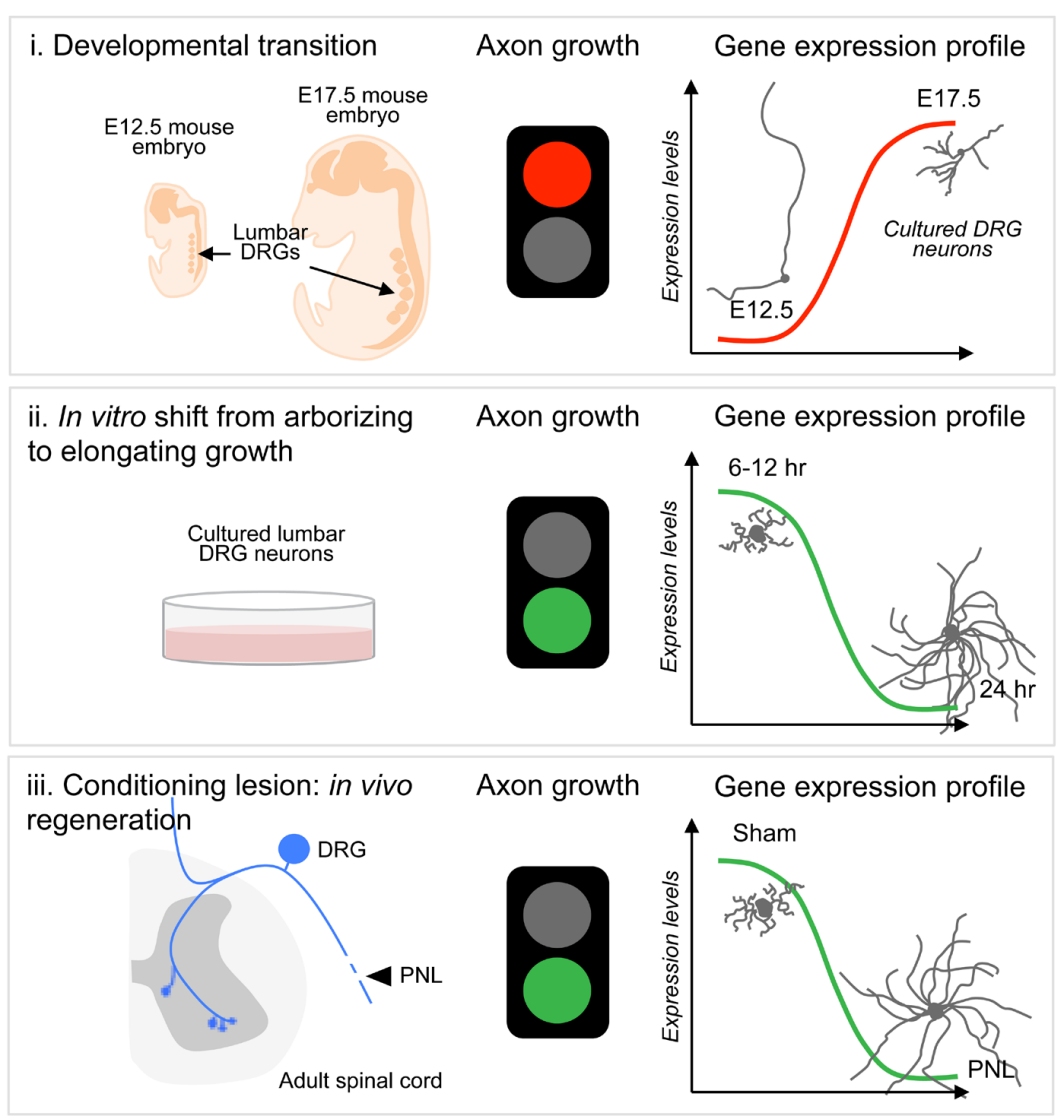
The transcriptional landscape of mouse dorsal root ganglia (DRG) neurons in both growth-competent and -incompetent states. DRG neurons have been instrumental in dissecting key molecular mechanisms of axon growth and regeneration failure. Whole transcriptome sequencing of DRG neurons from different stages of axon growth, including a developmental transition from axon growth to synapse formation, a shift from arborizing to elongating growth, and axon regeneration after a peripheral nerve lesion (PNL), has identified novel negative regulators of axon growth and regeneration, thus expanding the number of targets that could be manipulated for therapeutic gain. E, embryonic day.

When the transcriptional landscape of mouse DRG neurons was explored in both growth-competent and -incompetent states at different developmental stages, *Cacna2d2*, the gene encoding the α2δ2 subunit of voltage-gated calcium channels ^75^, was identified as a developmental switch that limits axon growth and regeneration ^27^ ( Figure 2). Interestingly, in these neurons, the developmental transition from a growth-competent (electrically dormant) to a transmitting (electrically active) phase is associated with a marked increase in the expression of genes that control synapse formation and synaptic transmission. Deletion or silencing of *Cacna2d2* in adult DRG neurons promotes axon growth *in vitro*. Pharmacological blockade of α2δ2 via systemic injection of gabapentinoids promotes regeneration of sensory axons after SCI in adult mice ^27^. Precisely how gabapentinoids enhance axon regeneration is unknown, but a mechanistic understanding is important, especially since these drugs are often used in humans to treat various neurological disorders, including neuropathic pain. Moreover, a multi-center cohort study found that motor recovery is improved in SCI individuals receiving gabapentinoids ^76^. Together, these data highlight the need to consider repurposing gabapentinoids as a novel treatment for CNS repair.

During development, a discrete number of transcription factors act as master regulators of gene expression. Among others, *Sox11* is highly expressed in many developing organs and its expression is turned off in adults. Whereas *Sox11* expression is not changed after CNS injury, its expression increases after peripheral injury, facilitating regeneration of injured peripheral nerves ^34, 77^. In normally non-regenerative cortical motor neurons, forcing *Sox11* expression enables sprouting and regeneration of corticospinal tract (CST) axons after unilateral pyramidotomy and cervical SCI, respectively ^28^. However, forced expression of *Sox11* in CST neurons impairs, rather than improves, skilled forelimb functions ^28^. Thus, improved axon regeneration does not necessarily predict that functional recovery also will improve. Intuitively, this makes sense since functionally significant axon regeneration is a multi-step repair process in which regenerating axons must re-establish proper synaptic connectivity in order to effectively integrate into existing or regrowing neuronal circuitry. Another study tested whether overexpression of *Sox11* or other ** master regulators of gene transcription can enhance regeneration of retinal ganglion cell (RGC) axons after optic nerve crush injury in adult mice. Only overexpression of *Sox11*, among the seven candidates tested, robustly increased RGC regeneration ^64^. Interestingly, a gene ontology analysis of transcriptomics data derived from RGC neurons overexpressing *Sox11* revealed that most genes that are suppressed by *Sox11* are associated with synaptic transmission ^64^, highlighting similarities with the α2δ2 findings described above. Together, these data suggest that genetic gain-of-function manipulations can rejuvenate adult neurons, enhancing their growth potential; however, these same manipulations may inadvertently impair synaptic function in the neural circuitry.

In a search for mechanisms underlying neural plasticity, a recent study profiled the transcriptome of sprouting intact neurons isolated from mice lacking Nogo receptor 1 (for example, a receptor for myelin-associated axon growth inhibitors) after incomplete SCI. In these mice, structural plasticity and regeneration of CST axons are enhanced in various CNS injury models ^78, 79^. The authors found that the lysophosphatidic acid (LPA) signaling modulators, LPPR1 and LPAR1, are intrinsic regulators of axon growth in corticospinal neurons. More specifically, LPPR1 overexpression or LPAR1 inhibition promoted collateral sprouting of intact CST axons and enhanced functional recovery after unilateral pyramitomy in adult wild-type mice. LPA is a bioactive lipid species derived from membrane phospholipids, and among the many cellular mechanisms that LPA signaling is known to affect (including oligodendrocyte maturation, myelination, astrocyte proliferation, and inflammation), synaptic transmission is also affected by LPA ^80^.

Single-cell RNA sequencing has emerged as a powerful technology that enables researchers to identify expression changes of thousands of genes in heterogeneous cell populations ^81, 82^. A recent study applied this technology to reveal DRG neuron heterogeneity and molecular dynamics after sciatic nerve transection ^40^. DRG neurons can be classified into several functionally distinct subtypes with very different gene expression patterns ^83– 85^. Such heretogeneity is reflected by the fact that injury to the peripheral branch of DRG neurons is often associated with mixed responses such as pain, cell death, plasticity, and axon regeneration. After segregating DRG neurons into different subtypes, weighted gene co-expression network analysis revealed injury-responsive gene modules with distinct expression patterns among the different subtypes. Interestingly, the cell death genes—programmed cell death-2 and neuron survival-like ISL LIM homeobox—were upregulated and downregulated, respectively, in a subset of non-peptidergic nociceptor neurons 3 days after injury ^40^. The fact that caspase-3 was upregulated in all injured subtypes suggests that these neurons may be more susceptible to cell death and therefore not able to regenerate. Indeed, a prerequisite for axon regeneration is that injured neurons survive. Dynamic changes in gene transcription in DRG neuronal subtypes were identified by completing an analysis of the transcriptome at 3 and 7 days after axotomy. Genes related to nervous system development, axonogenesis, regulation of metabolic process, and actin cytoskeleton reorganization were gradually upregulated in large myelinated neurons ^40^. In contrast, many genes related to learning or memory or nucleus organization were downregulated in these neurons ^40^, further implicating gene inactivation as an important regulator of axon growth programs (see above).

### Kinomics

A phenotypic screen of kinase inhibitors (that is, kinomics) combined with machine learning identified the ribosomal S6 kinase 1 (S6K1) as a negative regulator of axon regeneration in rodents ^42^. In *C. elegans*, ribosomal S6 kinase loss of function elicits new axon growth cone formation after injury and accelerates axon elongation ^86^. *In vitro*, S6K1 inhibition enhances growth of primary mouse hippocampal neurons. *In vivo* administration of a selective S6K1 inhibitor (for example, PF-4708671) promotes regeneration of CST axons into and beyond the lesion site in a model of cervical SCI. Functional recovery also is achieved in SCI animals treated with PF-4708671. The benefits of inhibiting S6K1, a known effector of the mammalian target of rapamycin (mTOR), conflict with data showing that mTOR is a positive regulator of axon regeneration in mammals ^16, 87– 89^. Hence, new kinomics data have enriched our understanding of molecular mechanisms of axon regeneration by showing that PI3K/mTOR signaling is negatively regulated by S6K1.

### Phosphoproteomics

Growth cones are specialized structures that are required during axon growth and regeneration ^90^. A better understanding of the signaling pathways that control growth cone activity may be necessary to gain control of axon growth, guidance, and regeneration as well as formation of neural circuits. Reversible protein phosphorylation is one of the most studied post-translational modifications. Phosphorylation of proline, serine, threonine, and tyrosine residues plays a crucial role in function, subcellular localization, and degradation of proteins, thus participating in various cellular processes, including signal transduction. A phosphoproteomic study of growth cone membranes isolated from postnatal day 1 rat forebrain identified 4596 phosphorylation sites from 1223 phosphoproteins ^41^. Of these phosphorylation sites, proline phosphorylation was the most represented. Analysis of the identified phosphoproteins suggested that cytoskeletal components and signaling proteins were the most abundant ^41^. Using a kinase-specific phosphorylation site prediction tool, the authors of this study revealed that proline phosphorylation was due to activation of the mitogen-activated protein kinase (MAPK) pathway ^41^. Of note, coordinated activation of highly conserved MAPK pathways is required for axon growth and regeneration ^68, 91– 93^. Strikingly, the most abundant phosphorylation site was an uncharacterized serine 96 of the growth-associated protein 43 (GAP-43), which is highly expressed during development and regeneration ^94– 97^. Of the different kinases involved in signal transduction, c-Jun N-terminus kinase (JNK) was responsible for numerous phosphorylated sites in the phosphoproteomic data set ^41^.

Another interesting study applied quantitative phosphoproteomics to study changes in protein phosphorylation in primary cerebellar granule neurons plated on growth-inhibitory chondroitin sulfate proteoglycans (CSPGs). Of the differentially phosphorylated proteins, phosphorylation increased on 41 peptides and decreased on 77 in neurons exposed to CSPGs ^98^. Cytoskeletal proteins were the top annotated category, representing 25 of the 118 phosphopeptides identified. Among these cytoskeletal proteins, 14 were of the actin family of cytoskeleton proteins. Cofilin, an actin depolymerization factor regulated by phosphorylation, plays an important role in growth cone behavior and neurite outgrowth ^99^. In neurons exposed to myelin-associated inhibitor, phosphorylation and inactivation of cofilin have been shown to be regulated via LIM kinase and slingshot phosphatase ^100^, contributing to axon growth inhibition and regeneration failure. Similarly, overexpression of the transcription factor serum response factor enhances axon regeneration through cytoplasmic localization and cofilin-mediated reactivation of actin dynamics in growth-inert retraction bulbs ^101^. The top three signaling pathways representing the 118 phosphopeptides were pyrimidine metabolism, p38MAPK pathway, and synaptic vesicle trafficking. Together, these results suggest that phosphoproteomics can serve as a powerful approach to unmask promising targets and signaling pathways to overcome regeneration failure in the adult CNS.

### Metagenomics

Successful axon regeneration may require a detailed understanding of genetic, proteomic, metabolic, and immunologic functions that occur in the body outside the nervous system. Metagenomics is a collection of high-throughput genetic analyses of transorganismal behaviors and the biosphere. Currently, most metagenomics studies focus on non-eukaryotic microbes, especially those found in the gastrointestinal tract (that is, the “gut”), to learn how these microbes affect organs and cells throughout the body, in both health and disease. Compelling data indicate that microbial metabolites, derived from gut microbes, directly affect the function of neurons and glia in the CNS. How or whether these metabolites will affect regeneration in the injured CNS has not been explored; however, robust and lasting changes in gut microbial communities do occur after a brain injury or SCI ^102– 105^. Injury-induced change in microbial populations is a potentially novel target for regulating the structure and function of injured neurons. Indeed, the magnitude and diversity of the microbial “payload” are remarkable—unique microbial genes outnumber mammalian genes by about 150 to 1 ^106^, and an impressive number of microbial enzymes and metabolites are already known to affect the metabolism and function of mammalian cells, including those in the immune and nervous systems ^107– 109^. Thus, it is easy to speculate that metagenomic and metabolomic techniques, when applied in the context of axon regeneration models, will reveal novel roles for microbes in affecting axon regeneration.

Although recent progress in the field of axon regeneration clearly illustrates the power of using omics-based approaches to reveal novel molecular mechanisms to target for therapeutic enhancement of axon growth, we believe that a better understanding of the mechanisms controlling presynaptic biogenesis, synaptic alignment, and connectivity will be necessary to rebuild injured neural circuits in a functionally meaningful way.

## Neuronal metabolism and mitochondrial transport

Promoting successful axon regeneration will likely require that we understand more than those genes and proteins that directly affect the physical structure of axons and synapses. Indeed, axon regeneration is a metabolically active, multi-step process. Omics technologies have revealed that optimal axon regeneration also depends on efficient mitochondrial transport and energy production in injured axons.

**Figure 3.**
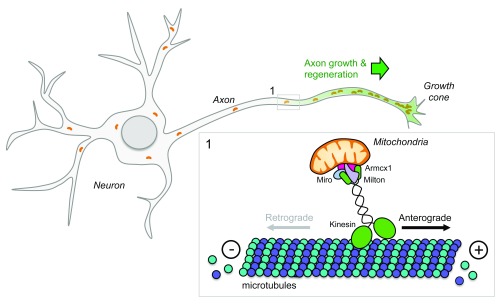
Mitochondria participate in axon regeneration. Mitochondria are actively transported to axons via axonal microtubules with their plus ends pointing toward the distal part and their minus ends facing the cell soma. Members of the kinesin family are responsible for mitochondria anterograde transport. Kinesin motors generate force by hydrolyzing adenosine triphosphate. Given that mitochondria provide energy for axonal functions (including active transport and membrane fusion), alteration of mitochondrial distribution along the axon leads to defects during development, maintenance, and regeneration of the nervous system.

Recent evidence indicates that enhancing mitochondrial transport promotes neuron survival and axon regeneration in experimental models of axotomy in worms ^110^ and mice ^25, 111^ ( Figure 3). Anterograde mitochondrial transport requires kinesin-1 motors, whereas dynein motors control retrograde transport back to the soma ^112– 114^. Live imaging of laser axotomized GABA motor neurons in mutant worms with an enhanced regenerative capacity has shown that mitochondrial density increases in regenerating axons and that regeneration can be enhanced further by experimentally boosting mitochondrial transport. Conversely, axon regeneration is poor in mutant worms with deficient mitochondrial transport. Mitochondrial localization to the axon is regulated in part by dual-leucine zipper kinase 1 (DLK-1) ^110^, an evolutionarily conserved intrinsic regulator of axon growth and regeneration in worms, flies, and mice ^92, 115– 118^.

Again, using the optic nerve injury model, high-throughput analysis of gene expression in RGC neurons has revealed that the armadillo repeat containing x-linked 1 (Armcx1) is a critical regulator of mitochondrial transport and plays a key role in promoting axon regeneration after optic nerve crush injury. Armcx1 localizes to mitochondria and interacts with components of the mitochondrial transport machinery, such as Miro 1 ^25^. Whereas Armcx1 overexpression enhances mitochondrial transport in mouse retinal explants and promotes RGC neuron survival and regeneration after optic nerve crush injury ^25^, its downregulation negatively impacts axon regeneration.

Adenosine triphosphate (ATP) is the major source of cellular energy produced by mitochondria. In injured CNS axons, mitochondria acutely depolarize, causing energy deficits along the injured axons ^111^. Recent data indicate that it is possible to reverse injury-induced energy loss and restore regenerative capacity in cultured mouse neurons. Indeed, overexpressing Miro 1 or knocking down the mitochondria-anchoring protein syntaphilin enhances mitochondrial transport and restores the energy balance in injured axons, leading to enhanced axon regeneration ^111^.

Using proteomics and bioinformatics techniques to analyze the injury response in axotomized RGCs, Belin *et al*. identified 12 signaling hubs, including several neuronal intrinsic regulators of axon growth and regeneration ^33^. The top three connected nodes are the tumor suppressor p53 ^119^, c-Myc ^120^, and Rictor. The authors focused on c-myc because of its role as master transcriptional regulator of several target genes that coordinate the *de novo* synthesis of new lipids and proteins that are needed for axon elongation. Indeed, forced expression of c-Myc in RGCs promotes neuron survival and regeneration after optic nerve crush injury ^33^. Although it is possible to manipulate oncogenes to achieve regenerative growth in CNS neurons in animal models, whether it is safe or prudent to do so in humans is questionable.

A recent study in *C. elegans* has shed some light on metabolic regulation controlling neuron repair after axotomy. O-linked β-N-acetylglucosamine (O-GlcNAc), a post-translational modification of serine and threonine residues of nuclear and cytoplasmic proteins, functions as a nutrient sensor and metabolic mediator by linking glucose metabolism to the hexosamine biosynthetic pathway. Twenty-four hours following laser axotomy *in vivo*, a decrease in O-GlcNAc levels promotes axon regeneration of either the anterior or posterior lateral microtubule neurons via ARK-1/AKT-1 signaling, using glycolysis as the primary source of energy ^121^. Blocking glucose transport or inhibiting glycolysis leads to axon regeneration failure in mutant worms with decreased O-GlcNAc levels ^121^. By contrast, increasing O-GlcNAc levels acts on mitochondrial function and enhances axon regeneration in *C. elegans* through FOXO/DAF-16–dependent mechanisms ^121^. These seemingly contradictory results may be explained by the fact that ** O-GlcNAc levels drive distinct branches of the insulin pathway to promote regeneration in worms.

The liver kinase B1 (LKB1) links cellular metabolism and energy homeostasis to cell polarity and growth ^122, 123^. LKB1 phosphorylates the central metabolic sensor AMPK, whose activation regulates cholesterol, lipid, and glucose metabolism ^123^. LKB1 overexpression in corticospinal neurons of adult mice was recently shown to promote long-distance regeneration of CST axons in experimental models of SCI ^31^. Also, systemic overexpression of LKB1 in mice causes descending serotonergic and tyrosine hydroxylase-positive axons to regrow into caudal segments of the injured spinal cord. Mechanistically, the AMP-activated protein kinase alpha, NUAK family SNF1-like kinase 1, and extracellular signal–regulated kinase act as effectors of LKB1 to promote axon growth and regeneration ^31^. Importantly, enhanced axon growth and regeneration in LKB1-overexpressing mice correlated with improved recovery of locomotor function ^31^.

Together, the above examples highlight the importance of achieving efficient mitochondrial transport and energy production in injured axons to fuel axon regeneration. Whether boosting neuronal metabolism and other metabolic pathways are sufficient to repair the injured CNS requires further investigation.

## Conclusions

During the last three years, novel candidates and combinatorial approaches that can promote structural plasticity, regeneration, and some degree of functional recovery have been identified ^27, 28, 31, 39, 124– 128^. To maximize chances to achieve functional recovery, however, axon regeneration, neuronal metabolism, synapse formation, and functional connectivity need to be spatially and temporally controlled to allow the establishment, refinement, and consolidation of essential neural circuitry ^23^. Thus far, data suggest that prolonged activation of neuron-intrinsic pathways causes defects in target innervation in several experimental injury models ^33, 129, 130^. Failure to re-innervate target neurons negatively impacts functional recovery and can cause neurobehavioral abnormalities that impair normal daily activities and thus quality of life. In addition, cardiovascular disease and autonomic dysfunction have become a growing concern for individuals with SCI ^131, 132^. Thus, turning off or reducing intrinsic axon growth ability together with cardiovascular rehabilitation, activity-based training, or other facilitators may indeed facilitate synapse formation, refinement, and consolidation of functional connectivity in the injured CNS. Several lines of evidence also suggest that adult regenerating axons can be guided toward specific target areas by providing chemoattraction ^133, 134^. Modulating astrocyte behavior to control synapse formation and elimination represents another intriguing direction for future studies ^135, 136^.

Several signaling pathways described above are conserved across different species. This will likely facilitate the translation of data obtained in smaller organisms and animal models to larger and more complex mammalian systems, including humans. Still, genetic variation exists within each model organism ^36, 137, 138^, so exploring the robustness of treatment strategies across different genetic backgrounds, within and between species, will be prudent before embarking on randomized clinical trials in humans. Lately, despite the generation of large omics data sets, a significant amount of information remains hidden. In our opinion, validation of omics approaches with stringent criteria and additional assays is an essential step to facilitate translation of breakthrough discoveries from the laboratory into clinical practice. Although there is no optimal strategy for integrating multi-omics data sets, more integration is likely to provide the most realistic picture about true biology. It is now possible to integrate data from transcriptomics, phosphoproteomics, and metabolomics. When combined with multi-layer omics ^139– 141^, the recent development of powerful computational methods ^142– 144^, machine learning and artificial intelligence ^145, 146^ will allow data mining and extracting principles and key biological information on a broad range of normal and disease conditions. Hence, automated inference methods should allow the rapid development and testing of new hypotheses and establish potential causal relationships in large data sets. As we enter a new era of regenerative medicine, we will be able to select combinations of treatment strategies for a personalized medicine approach to aid CNS repair.
